# Effects of Annealing on Microstructure and Mechanical Properties of Metastable Powder Metallurgy CoCrFeNiMo_0.2_ High Entropy Alloy

**DOI:** 10.3390/e21050448

**Published:** 2019-04-30

**Authors:** Cui Zhang, Bin Liu, Yong Liu, Qihong Fang, Wenmin Guo, Hu Yang

**Affiliations:** 1 State Key Laboratory for Powder Metallurgy, Central South University, Changsha 410083, China; 2 College of Mechanical and Vehicle Engineering, Hunan University, Changsha 410082, China; 3 Yuanmeng Precision Technology (Shenzhen) Institute, Shenzhen 518055, China

**Keywords:** powder metallurgy, high entropy alloy, microstructure, precipitation strengthening, mechanical properties

## Abstract

A CoCrFeNiMo_0.2_ high entropy alloy (HEA) was prepared through powder metallurgy (P/M) process. The effects of annealing on microstructural evolution and mechanical properties of P/M HEAs were investigated. The results show that the P/M HEA exhibit a metastable FCC single-phase structure. Subsequently, annealing causes precipitation in the grains and at the grain boundaries simultaneously. As the temperature increases, the size of the precipitates grows, while the content of the precipitates tends to increase gradually first, and then decrease as the annealing temperature goes up to 1000 °C. As the annealing time is prolonged, the size and content of the precipitates gradually increases, eventually reaching a saturated stable value. The mechanical properties of the annealed alloys have a significant correspondence with the precipitation behavior. The larger the volume fraction and the size of the precipitates, the higher the strength and the lower the plasticity of the HEA. The CoCrFeNiMo_0.2_ high entropy alloy, which annealed at 800 °C for 72 h, exhibited the most excellent mechanical properties with the ultimate tensile strength of about 850 MPa and an elongation of about 30%. Nearly all of the annealed HEAs exhibit good strength–ductility combinations due to the significant precipitation enhancement and nanotwinning. The separation of the coarse precipitation phase and the matrix during the deformation process is the main reason for the formation of micropores. Formation of large volume fraction of micropores results in a decrease in the plasticity of the alloy.

## 1. Introduction

Designing strong and ductile metals has been among the most ambitious goals for metallurgists [1,2,3,4]. During the past decade, a new concept of high entropy alloys (HEAs), which is broadly defined based on the high entropy effect of alloys with multi-components, has attracted great attention due to their unique superior properties, such as high strength, good ductility, high thermal stability, corrosion and oxidation resistance [5,6,7]. The most studied HEAs can be divided into two categories: one is FCC HEAs, with Fe, Co, Ni, Cr, Mn and other transition elements as main components, and the other is BCC HEAs, with refractory metals as main components [8,9,10]. Previous studies have demonstrated that FCC structured HEAs generally exhibited high tensile elongation but relatively low yield strength, while BCC alloys are the opposite, although some relatively ductile BCC refractory HEAs have been reported [11,12,13].

Simultaneous improvement of strength and ductility of alloys has been very challenging. Although single-phase FCC high-entropy alloys struggle to meet the high strength requirements of engineering materials, such alloys exhibit high work hardening rates and uniform deformation behavior [14,15]. These characteristics make high-entropy alloys prone to be an excellent composite matrix. Yang et al. [1] reported that the FCC based (CoFeNi)86-Al7Ti7 alloy can be significantly strengthened by the L12 multicomponent intermetallic nanoparticles and exhibit superior strength of 1.5 GPa and ductility as high as 50% in tension at ambient temperature. Liu et al. [16] reported that the FCC CoCrFeNiNbx can be significantly strengthened by the Nb-enriched Laves phase with the HCP structure. He et al. [17] reported that the addition of Al to the CoCrFeNiMn alloy can form a BCC phase, resulted in a high tensile strength up to 1174 MPa. The above studies show that the formation of high hardness reinforcing particles is an effective way to prepare high performance high entropy composite alloys.

Presently, different kinds of topologically close-packed (TCP) phases, such as σ, μ, Laves, etc. are observed in HEAs [18,19]. Adding this reinforcing phase to the FCC high entropy alloys can significantly increase their strength, but at the same time significantly reduce their plasticity [20]. Based on the precipitation strengthening effect, the most important thing is to control the morphology and distribution of the precipitated phase, and then to alleviate their harmful effects on ductility. As reported, the size and distribution of the reinforcing phase is very sensitive to heat treatment conditions. Gwalani et al. [21] reported that the L12 Ni_3_ (Ti, Al) nano-precipitates in the Al0.3CrFeCoNi alloy can only be stably present in the temperature range of 500–600 °C. As the temperatures above ~700 °C, these precipitates are dissolved and replaced by coarser ordered B2 precipitates. Liu et al. [14] reported that the precipitated nano σ phase formed during the heat treatment at 850–900 °C can simultaneously increase the strength and plasticity of the CoCrFeNiMo_0.3_ alloy. Ming et al. [22] reported that the Cr15Fe20Co35Ni20Mo10 HEA exhibit a superb strength–ductility combination by precipitation hardening of nanoscale precipitates of Mo-rich μ phase. It is suggested that a reasonable heat treatment process is essential for the formation of nano-precipitates, generally resulting in the improved comprehensive mechanical properties of alloys.

Powder metallurgy technology is one of the effective methods to avoid component segregation and obtain high performance composite materials. There have been many reports on powder metallurgy high entropy alloys [23,24,25]. Due to the higher cooling rate during the preparation process, these alloys typically exhibit an equiaxed microstructure. Presently, the effects of precipitation strengthening on the mechanical properties of CoCrFeNi based HEAs prepared by powder metallurgy have not yet been reported.

In the present research, a CoCrFeNiMo_0.2_ high entropy alloy was prepared by P/M process. A systematic study on precipitation behavior of the reinforcing phase is obtained. The effects of annealing on microstructure and mechanical properties of powder metallurgy CoCrFeNiMo_0.2_ high entropy alloy were also discussed.

## 2. Experimental Procedures

The CoCrFeNiMo_0.2_ spherical powders were obtained by an atomization process with high purity Fe Co Cr Ni and Mo raw materials. These raw materials were firstly melted in a vacuum furnace. And then, the melt dropped through a ceramic tube and was atomized in high purity Ar with an atomization pressure was 4 MPa. The chemical composition and oxygen content of the atomized powders was characterized by chemical methods and the fusion method on a Leco O/N analyzer (LECO TCH 600) respectively.

Subsequently, the CoCrFeNiMo_0.2_ alloy was prepared by a hot extrusion process with the atomized powders. The dimensions of the stainless-steel mold used in the hot extrusion process is *d*60 × 150 mm^3^. The powder is first loaded into a stainless steel can, pre-heated at 1473 K for 60 min, and sealed under vacuum. The enclosed powders were immediately subjected to hot extrusion with an extrusion ratio of 6 and a velocity of ~10 mm/s on a 2500 T hydraulic press. The as-extruded alloy was annealed at different temperatures range from in the range between 700 and to 1000 °C in vacuum for different times range from 2 to 72 h respectively, and then water quenched.

Tensile samples with *d*4 × 15 mm^3^ were prepared with the as-extruded and annealed HEA alloys along the extrusion direction (ED). Microstructures of these alloys were analyzed using a field emission scanning electron microscope (FESEM) (FEI Nova Nano-230, Hillsboro, OR, USA) equipped with an electron backscattered diffraction system (EBSD). The size and volume fraction of the precipitated phase were obtained by image analysis using Imagepro software. Phase structures were identified by an X-ray diffractometer (XRD) (Rigaku D/MAX-2250, Tokyo, Japan) with a Cu/Ka radiation. Tensile tests were performed with a loading strain rate of 10^−3^/s on an Instron 3369 testing machine at room temperature. The standard bright-field images and diffraction patterns were obtained using a transmission electron microscope (Tecnai G2 F20 S-TWIN, FEI, Hillsboro, OR, USA).

## 3. Results and Discussion

### 3.1. Microstructures

**Figure 1:**
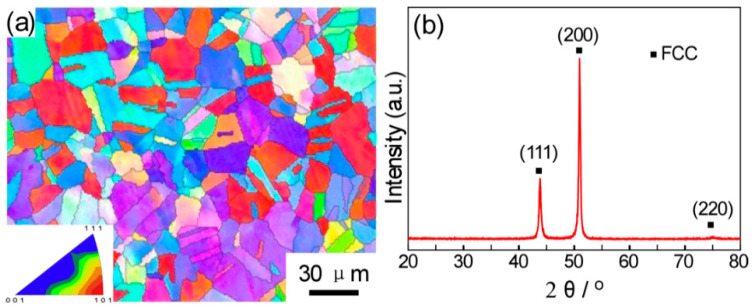
(**a**) IPF map of the P/M CoCrFeNiMo_0.2_ high entropy alloy (HEA), (**b**) XRD patterns of the P/M CoCrFeNiMo0.2 alloy.

Figure 1a shows the inverse pole figure (IPF) of the as-extruded CoCrFeNiMo_0.2_ HEA. It is obvious that the extruded alloy exhibits an equiaxed grain structure with an average grain size of approximately 20 μm. The relative density of the as-extruded alloy is approximately 99.5%. The consolidated microstructure is fully recrystallized, indicating that recrystallization occurred during and after the extrusion. Figure 1b shows the XRD patterns of the P/M CoCrFeNiMo0.2 HEA, where the alloy shows clearly a single FCC structure.

**Figure 2:**
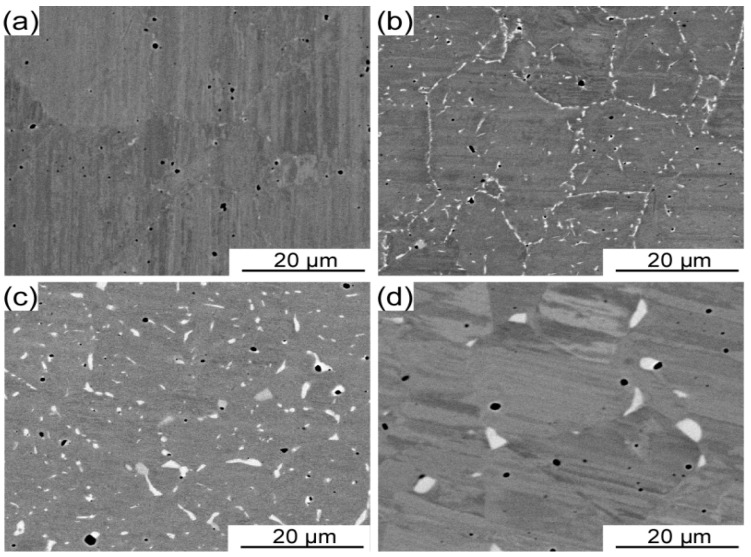
SEM images of the P/M CoCrFeNiMo_0.2_ HEAs annealed at different temperatures for 72 h. (**a**) 700 °C, (**b**) 800 °C, (**c**) 900 °C, and (**d**) 1000 °C.

Figure 2 demonstrate the microstructures of the Co Cr Fe Ni Mo 0.2 HEAs annealed at different temperatures in the range of 700–1000 °C for 72 h respectively. These annealed alloys are mainly composed of distinct grey matrix, black pores and white areas. It is apparent from Figure 2a to d that the size of these white areas gradually increases as the annealing temperature increased. As shown in Figure 2b, white areas generally appear at the grain boundaries and the size is less than 1 μm. As the annealing temperature increased up to 1000 °C, the size of these white areas is rapidly coarsened to 3–5 microns. In addition, the volume fraction of these white areas tends to increase gradually first and then decrease, as the annealing temperature goes up to 1000 °C.

**Figure 3:**
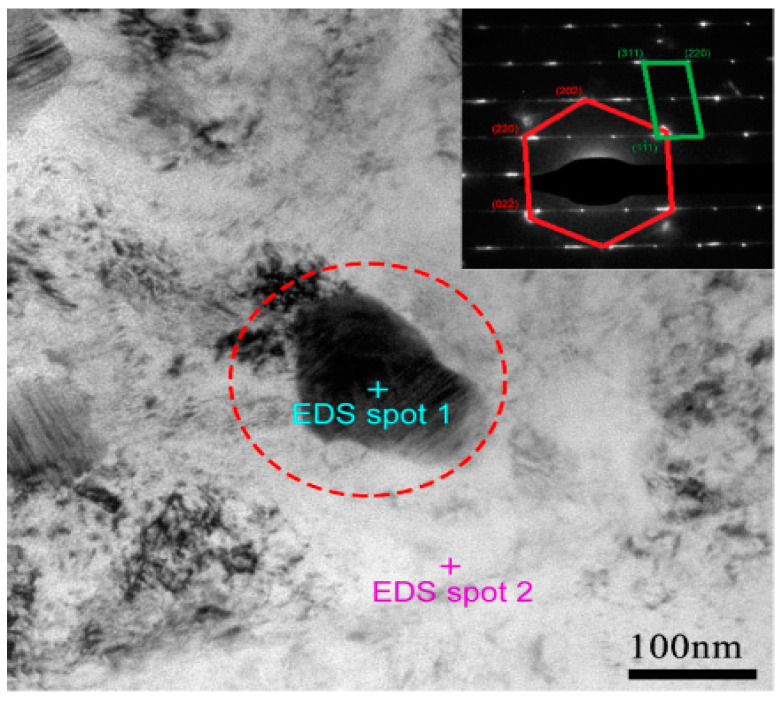
TEM image of the P/M CoCrFeNiMo_0.2_ HEA annealed at 700 °C for 72 h.

In order to further identify the crystal structure of the P/M CoCrFeNiMo_0.2_ HEA, we performed the TEM analysis on the precipitated phase. It is reported that the σ phase and μ phase in the CoCrFeNiMo_x_ alloy systems is corresponding to the stoichiometric (Cr,Mo)(Co,Fe,Ni) and (Mo,Cr)_7_(Co,Fe,Ni)_6_ respectively [26,27]. The EDS analysis results in Table 1 clearly indicates that the chemical composition of the precipitates contains a high concentration of Mo, which is very close to the σ phase reported by Shun et al. [27,28]. The selected electron diffraction pattern embedded in the upper right corner of Figure 3 also confirms that the white precipitates are σ phase. This result is also consistent with the calculated pseudo binary (CoCrFeNi)-Mo phase diagram [14].

**Figure 4:**
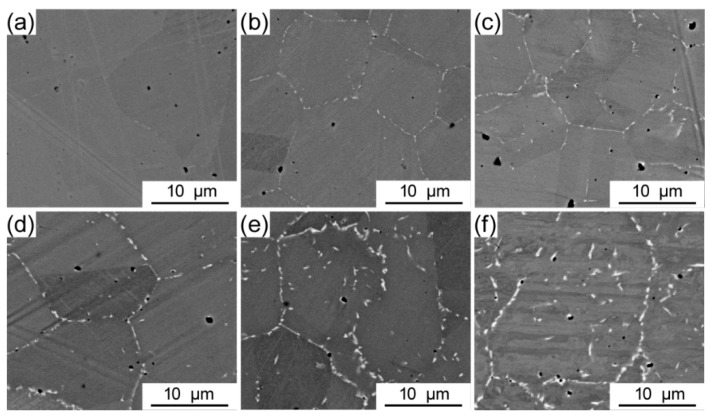
SEM images of the P/M CoCrFeNiMo_0.2_ HEAs annealed at 800 °C for different times: (**a**) 2 h, (**b**) 4 h, (**c**) 8 h, (**d**) 16 h, (**e**) 48 h, and (**f**) 72 h.

The microstructures of the CoCrFeNiMo_0.2_ HEAs annealed at 800 °C for various times are illustrated in Figure 4. It is apparent that prolongation of the annealing time promotes the precipitation of the σ phase. As shown in Figure 4b, σ phase began to appear at the grain boundary of the matrix as the annealing time was 4 h. σ phase grows with annealing time and changes its morphology.

**Figure 5:**
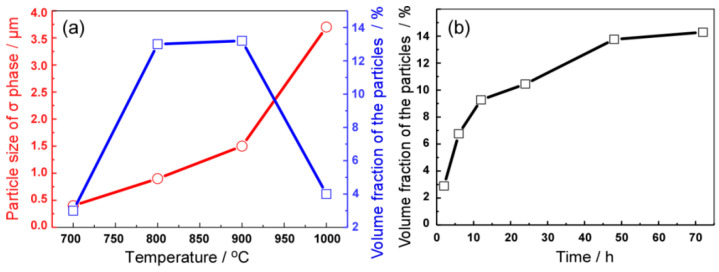
(**a**) Variation of the average size and volume fraction of σ precipitate with annealing temperatures; (**b**) Variation of the volume fraction of σ precipitate with annealing times at 800 °C.

Figure 5 shows a statistical analysis on the variation of the size and volume fraction of the σ phase with the annealing temperature and time. As shown in Figure 5a, the size of the σ phase is less than 0.5 μm as the annealing temperature at 700 °C. As the temperature increases, the size of the σ phase rapidly grows to the micron level. When the annealing temperature increases to 1000 °C, the size of the σ phase reaches to 3.7 μm. As seen from Figure 5b, the content of the precipitated phase is also closely related to the annealing time. As the annealing time is prolonged, the volume fraction of the precipitates gradually increases, eventually reaching a saturated stable value. Therefore, obtaining a uniformly dispersed nanoprecipitate phase in the CoCrFeNiMo_0.2_ HEA is strictly controlled by the heat treatment process.

### 3.2. Mechanical Properties

**Figure 6:**
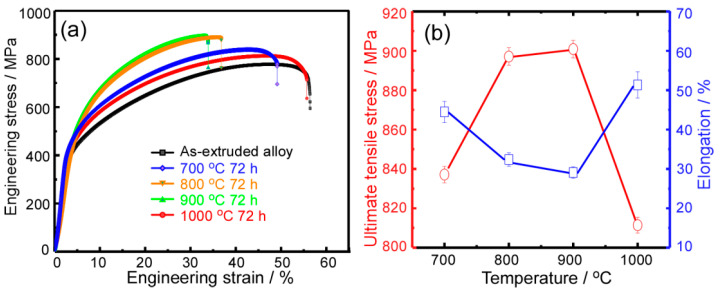
(**a**) Room-temperature engineering stress-strain curves for these HEAs annealed at different temperatures; (**b**) variation tendency of ultimate tensile strength and elongation of the annealed alloys with at different annealing temperatures.

Figure 6a shows the engineering stress-strain curves of the as-extruded and annealed CoCrFeNiMo_0.2_ HEA at different temperatures for 72 h. The evolution of tensile strength and plasticity with annealing temperature was also statistically calculated in Figure 6b. A typical elasto-plastic deformation behavior is remarked. All these annealed alloys exhibited a long work hardening stage. The yield strength, ultimate tensile strength and elongation to failure of the as-extruded CoCrFeNiMo_0.2_ HEA were about 400 MPa, 781 MPa and 55.6% respectively. As the annealing temperature increases from 700 °C to 900 °C, it is apparent that the yield strength and ultimate tensile strength of these annealed alloys were gradually improved. However, the ductility of these alloys has also dropped significantly. Since the annealing temperature increases up to 1000 °C, the yield strength and ultimate tensile strength of the alloy is significantly decreased, while the plasticity is correspondingly increased to as high as approximately 65%. Although such a tradeoff relationship between the strength and plasticity is conventional, the comprehensive mechanical properties of these annealed CoCrFeNiMo_0.2_ alloys are significantly better than those of the as-extruded one.

**Figure 7:**
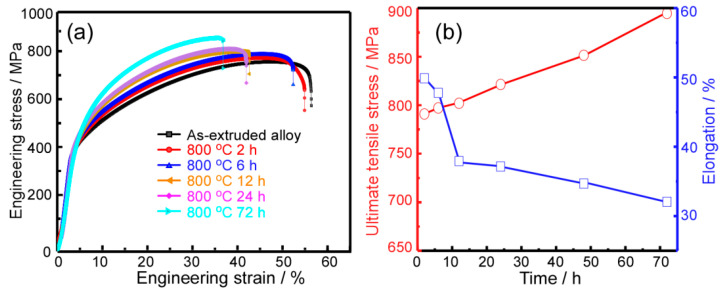
(**a**) Room-temperature engineering stress-strain curves for the HEAs annealed at 800 °C for various times; (**b**) variation tendency of ultimate tensile strength and elongation of the annealed HEAs at different annealing times.

Figure 7a shows the engineering stress-strain curves of the as-extruded and annealed CoCrFeNiMo0_.2_ HEA at 800 °C with different annealing times. The evolution of tensile strength and plasticity with annealing time was also statistically calculated in Figure 7b. With the prolongation of annealing time, the yield strength and ultimate tensile strength of these annealed alloys gradually increase, while the plasticity decreases gradually. Combined with the Figure 5, it is obvious that the evolutionary trend of the mechanical properties of the annealed alloys has a significant correspondence with the precipitate behavior of σ phase. The enhanced yield strength and ultimate tensile strength is mainly attributed to the high content and grain growth of the σ phase. The larger the volume fraction and the size of the σ phase, the higher the strength while the lower the plasticity of the alloy.

**Figure 8:**
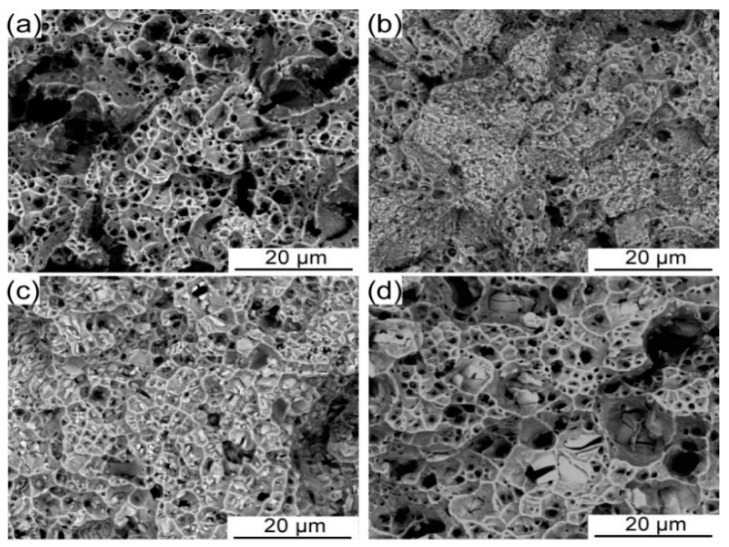
The morphologies of the fractured surface of the P/M CoCrFeNiMo0.2 HEAs annealed at different temperatures for 72 h. (**a**) 700 °C, (**b**) 800 °C, (**c**) 900 °C, and (**d**) 1000 °C.

Figure 8 shows the fracture surface of CoCrFeNiMo_0.2_ HEA annealed at different temperatures in the range from 700 °C to 1000 °C for 72 h. The presence of a large number of dimples on the fracture surface indicates that these alloys have good deformability [29]. As the annealing temperature increases, it can be found that the size of the dimples becomes significantly bigger. Simultaneously, obvious signs of broken phase of the precipitated phase can be found at the bottom of the dimple. It is found that the separation of the precipitation phase and the matrix during the deformation process is the main reason for the formation of micropores. Due to the difference between the elastic modulus, stress concentration is likely to occur at the interface between the precipitated phase and the matrix during the deformation process. The size of the micropores increases with this coarse precipitated phase, indicating crack propagation in the localized region of the alloy was significantly affected by these precipitated phase. Formation of large volume fraction of micropores results in a decrease in the plasticity of the alloy.

**Figure 9:**
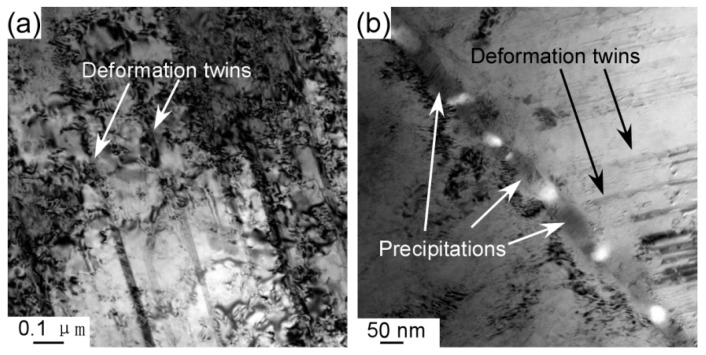
TEM bright-field images of the fractured CoCrFeNiMo0.2 HEA annealed at 700 °C for 72 h: (**a**) showing the nano twinning and (**b**) showing the interaction of dislocations with nanoscale σ precipitates.

Figure 9 illustrates the TEM bright-field images of the annealed CoCrFeNiMo0.2 alloy after tensile deformation. It can be seen that a large number of deformed twins formed during the tensile deformation process. The interaction of the precipitates, deformed twins and grain boundary results in the pileup of dislocations. The σ precipitates did not undergo plastic deformation during the dislocation slip, resulting in high strength and work hardening rate of the alloy. As can be seen from Figure 9b, the nanoprecipitate phase preferentially precipitates at the grain boundary. In addition, deformed twins also play an important role in improving the strength and toughness of the alloy. The twin boundaries have a significant hindrance to the slip of dislocations. Thus, these annealed CoCrFeNiMo_0.2_ HEAs plastically deform via dislocation gliding and nanotwinning, significantly enhancing strain hardening capability.

## 4. Conclusions

In the present research, a CoCrFeNiMo_0.2_ HEA is prepared using P/M method. The P/M HEA was annealed at different temperatures (700–1000 °C) for different times (2–72 h). The following conclusions are drawn:

1. The P/M CoCrFeNiMo_0.2_ HEA has a metastable FCC single-phase microstructure. During annealing, σ phase enriched with Mo and Cr precipitates in the grains and at the grain boundaries. As the temperature increases from 700 °C to 1000 °C, the size of the precipitates grows apparently. The volume fraction of the precipitates tends to increase gradually as the annealing temperature up to 900 °C and then decrease at 1000 °C. At 800 °C, the volume fraction of the precipitates gradually increases as the annealing time is prolonged, eventually reaching a saturated stable value about 14%.

2. The comprehensive mechanical property of the annealed CoCrFeNiMo_0.2_ HEAs has a significant correspondence with the precipitates. The larger the volume fraction and the size of the precipitates, the higher the strength and the lower the plasticity of the HEA. Nearly all the annealed HEAs exhibit good strength–ductility combinations due to the significant precipitation enhancement and nanotwinning.

## Figures and Tables

**Table 1:** EDS analysis results for the P/M CoCrFeNiMo_0.2_ HEA annealed at 700 °C for 72 h (Spots in Figure 3).

Location	Chemical Composition (at.%)				
	Mo L	Cr K	Fe K	Co K	Ni K
EDS spot 1	34.56	18.03	20.38	17.53	9.49
EDS spot 2	6.18	22.08	25.71	24.51	21.52
